# Improving Collaboration by Standardization Efforts in Systems Biology

**DOI:** 10.3389/fbioe.2014.00061

**Published:** 2014-12-08

**Authors:** Andreas Dräger, Bernhard Ø. Palsson

**Affiliations:** ^1^Systems Biology Research Group, Department of Bioengineering, University of California, San Diego, La Jolla, CA, USA; ^2^Cognitive Systems, Center for Bioinformatics Tübingen (ZBIT), Department of Computer Science, University of Tübingen, Tübingen, Germany

**Keywords:** model formats, modeling guidelines, ontologies, model databases, network visualization, software support

## Abstract

Collaborative genome-scale reconstruction endeavors of metabolic networks would not be possible without a common, standardized formal representation of these systems. The ability to precisely define biological building blocks together with their dynamic behavior has even been considered a prerequisite for upcoming synthetic biology approaches. Driven by the requirements of such ambitious research goals, standardization itself has become an active field of research on nearly all levels of granularity in biology. In addition to the originally envisaged exchange of computational models and tool interoperability, new standards have been suggested for an unambiguous graphical display of biological phenomena, to annotate, archive, as well as to rank models, and to describe execution and the outcomes of simulation experiments. The spectrum now even covers the interaction of entire neurons in the brain, three-dimensional motions, and the description of pharmacometric studies. Thereby, the mathematical description of systems and approaches for their (repeated) simulation are clearly separated from each other and also from their graphical representation. Minimum information definitions constitute guidelines and common operation protocols in order to ensure reproducibility of findings and a unified knowledge representation. Central database infrastructures have been established that provide the scientific community with persistent links from model annotations to online resources. A rich variety of open-source software tools thrives for all data formats, often supporting a multitude of programing languages. Regular meetings and workshops of developers and users lead to continuous improvement and ongoing development of these standardization efforts. This article gives a brief overview about the current state of the growing number of operation protocols, mark-up languages, graphical descriptions, and fundamental software support with relevance to systems biology.

## 1 Introduction

Since its emergence in the 1960s systems biology has always been tightly related to the availability of powerful computational resources. While at the beginning of research in the field and its applications quick and simple script-based solutions were sufficient, the bar for publication and review has been drastically raised (Sauro et al., 2003). It has been realized that individual scripts, which are specific to certain computational environments and that are not very reproducible are of small benefit for the scientific community and progress of the field (Lloyd et al., 2004). The development of standardized data formats, models, and computational methods have paved the way toward the evolution and maturation of systems biology into a main-stream field of research (Macilwain, 2011). Sufficient annotation and metadata of models, experiments, and other data enhance the reproducibility of results (Wolstencroft et al., 2011). For individual areas of research, different models are required, hence different standards for their encoding. Research in constraint-based modeling (Bordbar et al., 2014) deals with the encoding of the stoichiometric matrix and flux bounds, whereas, dynamic metabolic modeling (Dräger and Planatscher, 2013a) is usually based on building ordinary differential equation systems, model calibration, and parameter estimation (Dräger et al., 2009a; Kronfeld et al., 2009; Dräger and Planatscher, 2013b). Spatial-temporal simulations require encoding three-dimensional geometries and partial differential equation systems (Moraru et al., 2008).

It can hence be observed that the modeling community in systems biology has diversified. One reason for this development is that main parts of funding for these standardization attempts originate from ambitious large-scale projects, each having has different requirements. These efforts include, for example, goal of specifically reconstructing all reactions in specific organisms, such as human or yeast, resulting in giant reaction networks (Duarte et al., 2007; Herrgård et al., 2008; Rolfsson et al., 2011; Thiele et al., 2013) or systematically representing the complete knowledge about biochemical reactions available today (Büchel et al., 2013a). Trans-European projects like SysMO (Booth, 2007) want to comprehensively record and describe dynamic molecular processes in unicellular microorganisms and to present all processes in the form of computerized mathematical models. The German Virtual Liver Network (Holzhütter et al., 2012) aims to mathematically explain all phenomena in the human liver across multiple cell types and levels of organization. The Physiome project attempts to achieve a full quantitative description of all physiological dynamics and functional behaviors of the intact human body (Hunter and Borg, 2003). The US BRAIN (Brain Research through Advancing Innovative Neurotechnologies) Initiative aims to support the development of new technologies for classifying the anatomical constituents for the brain and to allow simultaneous recording from an unprecedented number of neurons simultaneously. The EUs Human Brain Project seeks to develop the infrastructure for creating computational models of brain regions at multiple scales on high-performance computing platforms (Shepherd et al., 1998; Markram et al., 2011; Kandel et al., 2013). Thereby, medical applications become increasingly important (Büchel et al., 2013b; Grillner, 2014).

Common to all these consortia is that with the increasing number of active researchers and collaborators the exchange, reproduction, and accessibility of models, data, and further information in specific online databases play a major role (Brazma et al., 2006; Schellenberger et al., 2010; Wolstencroft et al., 2011; Yu et al., 2011; Chelliah et al., 2013). Just like the documentation of source code, the careful annotation of models and data are also necessary to achieve a fruitful collaboration. The more meta information that is provided, the easier the model can be comprehended, modified, simulated, and analyzed (Waltemath et al., 2013). The use of standard formats is highly recommended for the publication of results even if not required by the prospective journal.

In addition, new fields and areas of application are emerging, for instance, pharmacometric models or synthetic biology (Endler et al., 2009; Galdzicki et al., 2011; Müller and Arndt, 2012). There is therefore no one-size-fits-all solution that would be equally suitable for all fields of research. The standardization community therefore needs to continuously catch up with these developments in the actual modeling community and to reinvent itself over and over again. Recent approaches have suggested to modularize modeling languages by introducing highly specialized *packages* for modeling aspects that can otherwise not be represented in the main data format (Chaouiya et al., 2013).

**Figure 1:**
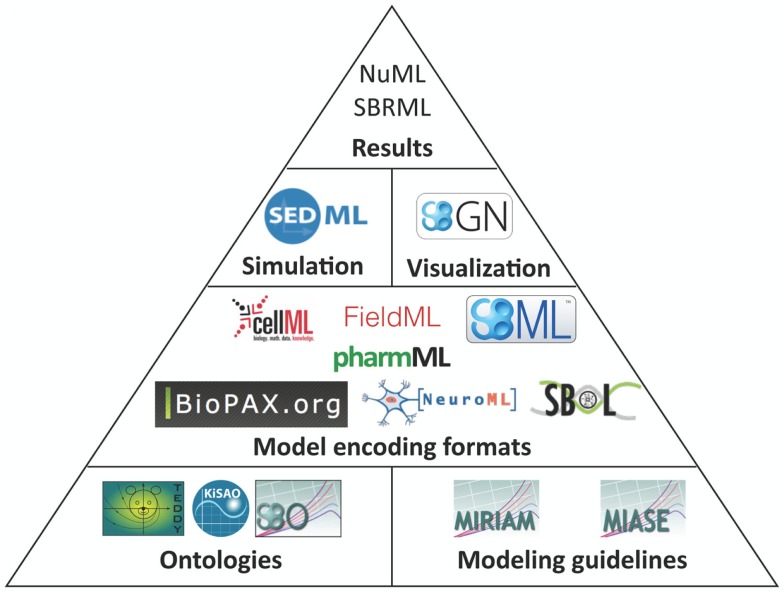
**Standards overview**. Hierarchically organized controlled vocabularies, so-called *ontologies* and modeling guidelines build the basis for model encoding formats. These formats can refer to terms from ontologies and their organization is in accordance with the modeling guidelines. Recommendations for a visual representation of models as well as the execution of individual models in numerical simulation or optimization are separated from the structural models. Numerical results can be encoded in further standard data formats.

The structure of how standards are defined has also matured. Brazma et al. (2006) describe that four steps are required for the development of a standard: (i) data and information need to be collected about the domain of interest that are relevant for an unambiguous transfer and interpretation as well as conceptual model design, (ii) the model needs to be formalized, (iii) an exchange format must be defined, and (iv) software support must be implemented. Nearly all modeling formats described in this article now follow this suggestion and are based on a minimum information requirement description (Taylor et al., 2008). These documents define what kind of information has to be stored in a respective model in order to guarantee that the model can be reused and understood by other researchers. In this way, the information requirement and the corresponding modeling standard are decoupled, exchangeable, and independent. The minimum information requirement is usually complemented with a specific ontology, i.e., a hierarchical collection of field-specific terms and their definitions (Courtot et al., 2011). These terms can be associated to model components and descriptions. In addition, elaborate and persistent annotation frameworks have been developed, which allow the modeler to precisely express, what individual model components are and how they are to be understood (Juty et al., 2012, 2013). The development of standards, minimal information requirements, and ontologies needs to be orthogonal to existing respective standards. Table 1 and Figure 1 give an overview about the relationship amongst various standards discussed in this article.

**Table 1:** **Standards with relevance for modeling in systems biology**.

	Model	Procedures	Results
Representation formats	BioPAX, CellML, NeuroML, PharmML, SBML (including extension packages), SBGN-ML, SBOL	SED-ML	NuML, SBRML
Graphical display	CellML visualization, SBGN, SBOL visual		
Minimal information requirements	MIRIAM	MIASE	
Mathematical semantics	SBO, MAMO	KiSAO	TEDDY
Biological semantics	MIRIAM	MIRIAM	MIRIAM

The structural representation of the model [for instance, SBML by Hucka et al. (2004) or CellML by Cuellar et al. (2006)], its application and analysis [SED-ML by Waltemath et al. (2011b)], its (graphical) display [SBGN guidelines by Le Novère et al. (2009)], and features should be accurately discriminated and encoded in distinct formats. Depending on the concrete modeling format, structural models can also include mathematical formulations, but not their interpretation framework (such as the algorithm to solve the model or the simulation end time). Recently, a new archive format has been proposed in order to link and distribute these independent modeling aspects all together in a single file (Bergmann et al., 2014).

Much effort has also been invested in software support and the creation of infrastructures for diverse standards. For each data format, a specific library has been implemented for reading and writing files as well as for manipulating components of the format in memory (Bornstein et al., 2008; Miller et al., 2010; Demir et al., 2013). Often, language-bindings for diverse programing environments are provided, but sometimes specific libraries have been developed in order to support certain programing languages (Dräger et al., 2011). These parsing libraries help developers to use and exploit the individual standards. Often these libraries provide interfaces to corresponding ontologies and controlled vocabulary annotations (Courtot et al., 2011). However, the interpretation, analysis, drawing, etc. of models cannot be facilitated by these libraries. Higher level software has been implemented to support model building, display, simulation, etc. (Deckard et al., 2006; Keller et al., 2013). Sometimes, this is done in the form of plug-ins to more general frameworks, and often there are diverse stand-alone or web-based tools for various purposes (König et al., 2012; Krause et al., 2013).

When the first XML- or OWL-based exchange formats for models were proposed, developers of existing software tools were often involved, and their individual software was adapted in order to fit the standard. Nowadays, with many standards being well established, software is specifically tailored with respect to the standards. The stringent elaboration and clear distinction between models, purpose, simulation, and annotation can also be a source of inspiration for young researchers who enter the field. In the long-term, using standard formats can lower the expenses for software development because they allow the reuse of existing tools in new applications. Moreover, with the many available tools for standard formats, less research time is needed for the interconversion of tool-specific files, making it much easier to collect information from diverse sources (Demir et al., 2010).

While international and national standardization bodies, such as OMG, W3C, IEEE, ANSI, IETF, DIN, etc., usually approve standards and release specifications, the situation is different in systems biology, where *de facto* standards are established by the scientific community (Brazma et al., 2006). The fast-moving nature and ongoing development of research makes this approach necessary.

However, keeping track of the growing number of model formats and standards for diverse purposes has become more and more difficult. This review article gives a broad overview of a wide range of currently existing modeling standards, formats, and online repositories, and a selection of software solutions for systems biology and related fields of research. The aim of this article is to highlight specific standards, their usability, and application in order to give the reader an up-to-date picture of model definition, encoding, and availability in systems biology.

## 2 Material and Methods

### 2.1 Modeling guidelines

Modeling formats give us the syntax of models (Juty et al., 2012). In order to enhance accessibility of data and to facilitate the reuse of models, several modeling guidelines have been proposed, which are discussed in this section. These guidelines are often called “Minimum Information of/for,” which should express that without at least this form of information optimal use and reproducibility of results cannot be guaranteed. More information can always be provided on top of the minimal requirements. The guidelines are hence a form of checklists that describe which kind of information to include and often go back to the idea of the MIBBI project (Minimal Information for Biological and Biomedical research) proposed by Taylor et al. (2008). The open biomedical ontologies (OBO) foundry^1^ maintains orthogonal (non-overlapping) collections of controlled vocabularies, which provide the semantics for models. The most well-known ontology is probably the gene ontology (GO) by (Ashburner et al., 2000).

#### 2.1.1 Minimum information required in the annotation of models

Reuse of models can be compromised if inconsistent identifier systems are used for individual components. For instance, when merging models, it is necessary to match overlapping components. If a molecule is identified as water in one model and as H_2_O in another such a matching is already difficult for automated procedures. To solve such problems, the minimum information required in the annotation of models (MIRIAM) guidelines has been proposed as a general model curation checklist (Le Novère et al., 2005). The MIRIAM registry (Laible and Le Novère, 2007) goes further and provides a connection between controlled vocabularies (Courtot et al., 2011) and formats, tools, and databases. Most modeling standards provide mechanisms to attach MIRIAM annotations to their components. These annotations are structured based on a subject-predicate-object scheme. Here, the subject is the identifier of the model element. The predicate is one of several predefined qualifiers, e.g., hasPart or is. The object should be a web resources pointing to an identifiers.org address (Juty et al., 2012, 2013), for instance http://identifiers.org/kegg.compound/C00001 for water. This Uniform Resource Identifier (URI) is therefore composed of the prefix identifiers.org/, the definition of the data collection (in this example kegg.compound) followed by the delimiter and finally the record identifier (here C00001). Using such an identifiers.org address instead of directly pointing to an entry in ChEBI (Brooksbank et al., 2013; Hastings et al., 2013), MetaCyc (Caspi et al., 2014), KEGG (Kanehisa and Goto, 2000), or any other of the more than 30 currently supported data collections has several advantages. Should the original resource location or address schema change, the identifiers.org site will point to the new location. identifiers.org also measures the uptime of mirror servers for identical records and preferably directs to the most reliable mirror.

#### 2.1.2 Minimum information about a simulation experiment

The minimum information about a simulation experiment (MIASE) project (Waltemath et al., 2011a) aims to unambiguously define how to reproduce the results of a model simulation. For stochastic models, the results should be within an acceptable small range from the original results, and for deterministic models, the results should be identical. This requirements checklist also supports the review process of scientific publications. Relevant ontologies (Courtot et al., 2011) for MIASE are the kinetic simulation algorithm ontology (KiSAO) that defines the method to use, the terminology for the description of dynamics (TEDDY), and the mathematical modeling ontology (MAMO).

#### 2.1.3 Ontologies

##### 2.1.3.1 Kinetic simulation algorithm ontology

The KiSAO gathers computational methods that can be used to simulate a model in a certain way (Courtot et al., 2011). It contains, for instance, definitions of several differential equation solvers for numerical calculations. Organizing these algorithms in a hierarchical structure allows tools to automatically select the most similar solver within their collection of implemented methods.

##### 2.1.3.2 SBO

The Systems Biology Ontology is a collection of terms that describe the structure of a model, its components, modeling frameworks, and processes (Courtot et al., 2011). By using terms from this ontology, the semantics of individual parts of a model can be made explicit. This is often of particular importance if elements can participate in processes where they can have multiple roles, such as catalysts or inhibitors.

##### 2.1.3.3 Mathematical modeling ontology

The recently developed ontology (MAMO, see http://bioportal.bioontology.org/ontologies/MAMO) has complemented and refined the *modeling framework* branch of SBO. Both ontologies are intended to cross link each other. While SBO mainly focuses on the entities and parameters in the model and describes the relationships among them, MAMO has been developed in order to precisely define and categorize *types* of mathematical models (e.g., *ODE*) and their characteristics (e.g., *discrete* vs. *continuous*) as well as types of readouts (such as *time-course analysis*) and variables (such as *dependent variable*).

##### 2.1.3.4 Terminology for the description of dynamics

The TEDDY defines a formal way to specify how the numerical results of a dynamic system behave when a simulation experiment is conducted (Knüpfer et al., 2006; Courtot et al., 2011). In this way, a machine-readable representation of such a description can be automatically generated upon simulation and be stored along with the model. When querying a database of numeric results, this terminology can help to find models with a desired behavior, such as ongoing oscillations.

### 2.2 Modeling formats

Reconstructing computational models based on a textual description in a publication can be difficult, because required information, such as a clear definition of the units of all components, can be lacking, the language might be imprecise or ambiguous, or a combined explanation of simulation procedure and actual model hamper the implementation of the model (Cooling, 2010; Dräger et al., 2010). In cases, where models are distributed in form of source code implemented for a specific run-time environment or programing language, executing these programs can be a challenge because of diverse dependencies to operating systems or required third-party libraries (Lloyd et al., 2004). In this section, we will discuss several formats that encode systems biological models in different ways with the aim to overcome this problem.

#### 2.2.1 Systems biology mark-up language

The Systems Biology Mark-up Language (Finney and Hucka, 2003; Hucka et al., 2003, 2004)^2^ is a hierarchical XML-based format consisting of several lists of components, such as compartments (finite spaces), (reactive) species, parameters (constants or variables), reactions with kinetic laws, user-defined functions and rules, events, units, and many more. SBML has been developed as a model exchange format that covers a wide range of modeling approaches used today (Hucka et al., 2004), including dynamic and steady-state metabolic networks as well as gene-regulatory and signaling networks (Lambeck et al., 2010; Vlaic et al., 2013). The term *reaction* should no longer be seen as a strict (bio-)chemical reaction. It is rather a process with inputs and outcomes. Specific annotation with SBO terms and MIRIAM identifiers clarify the purpose of all elements. The reactions implicitly define a differential equation system, whose explicit structure needs to be assembled at simulation time or prior to simulation. The rationale behind this design decision is that the same model can be interpreted in terms of a different modeling framework, such as stochastic simulation, etc.

The libraries libSBML (Bornstein et al., 2008) and JSBML (Dräger et al., 2011) facilitate the implementation of import and export functions of SBML models in customized software solutions. While libSBML provides bindings to a large variety of programing languages based on the wrapper generator SWIG (Beazley, 1996), the JSBML library has been specifically developed for the platform-independent Java™ language. Both libraries strive to attain a high degree of compatibility. Specific API libraries have also been implemented for working with SBML under MATLAB™ (Keating et al., 2006) and Mathematica™ (Shapiro et al., 2004, 2007).

It has been recognized that the interpretation and simulation of SBML models can be quite challenging and that different simulation environments can yield divergent results on identical input files (Bergmann and Sauro, 2008). For this reason, a comprehensive test suite of manually created SBML models has been established including reference results. This test suite can be used as a benchmark test case for simulation routines.

SBML handles the increasing diversification of modeling approaches and community requirements with the development of several specific and orthogonal packages, which can be used in addition or separately from the core format. The following extension packages have already been released: hierarchical model composition (comp) (Smith et al., 2013b), flux balance constraints (fbc) (Orth et al., 2010; Bergmann and Olivier, 2013), three-dimensional arrangement of elements in diagrams (layout) (Gauges et al., 2006), and qualitative relationships (qual) (Chaouiya et al., 2013). Draft specifications are available for the following extensions: arrays, sampling of values from statistical distributions (distrib), dynamic creation and destruction of structures during a simulation (dyn), grouping of elements (groups), entity pools with multiple states and complex composition of species (multi), drawing graphical representations of a model (render), indication of those model elements that are changed by packages (req), and spatial processes and geometries (spatial). For an up-to-date list and more detailed explanation of available extension packages, see http://sbml.org/Community/Wiki.

#### 2.2.2 CellML

The XML-based model storage and exchange format CellML^3^ has been developed for the IUPS Physiome project with the aim to facilitate reuse of models or their components in a software-independent manner (Lloyd et al., 2004; Cooling, 2010). CellML eases the creation of new models based on parts of existing models and hence accelerates the cumbersome model building process (Cooling et al., 2010). CellML models contain structural information about the organization of the model (components, connections, and units), mathematical equations (arbitrary MathML) to quantitatively describe biological processes, and metadata that link model components to online resources. An important design feature of CellML allows components and parameters to be shared across models via import statements and well-defined interfaces. This also allows users to structure their models into multiple files, similar as can be done with HTML pages, and increases reusability of individual black-box models, but also requires a strict decoupling of components. CellML uses RDF tags for semantic annotations and allows for hierarchical groupings of components. A set of software tools is available to edit CellML models, including an API implementation (Miller et al., 2010) or the graphical modeling environments OpenCell (Lloyd, 2013) and OpenCOR (Nickerson et al., 2013). CellML can be inter-converted from and to SBML and to the scripting language Antimony (Schilstra et al., 2006; Smith et al., 2013a). The rates of change of all components are explicit in CellML. When adding components or connections to a model, these rates of change would need to be updated. With the help of interfaces modelers can avoid this cumbersome update process (Cooling, 2010).

#### 2.2.3 FieldML

FieldML^4^ is an XML-based model interchange standard, which has been developed with a focus on the euHeart and Physiome projects and is currently available in version 0.5 (Britten et al., 2013). The main purpose of the format is to encode geometric models in explicit or implicit mathematical form with respect to biological and medical phenomena with spatial-temporal variation, such as the simulation of power fields and gradients. FieldML focuses on fields over multiple discrete indices and multivariate fields with discrete or continuous variables as well as interpolation functions. With these approaches, it is possible to model muscle contraction as part of cardiac mechanics, blood flows, and other multi-scale processes. Other applications include the modeling of patient-specific clinical images with the help of specific annotations and fitting of models to fields. Similar approaches are also planned for the spatial extension for SBML (Schaff et al., 2013). A powerful C++ API with wrappers for Java, Fortran, and Python as well as a software plug-in for the physiome model repository (PMR) support FieldML and provide several high-level functions for model building and simulation (Yu et al., 2011). Version 0.5 already includes model composition over multiple files and data sources.

#### 2.2.4 BioPAX

The motivation for the creation of the BioPAX^5^ format (Biological Pathway Exchange) was the aim to unify the various co-existing pathway encoding formats of numerous online databases (Demir et al., 2010). This format is intended to facilitate the communication between diverse software systems and also serves as a common knowledge representation of pathways. With BioPAX the structure of metabolic, signaling, and gene-regulatory pathways can be encoded, including relationships between elements (such as genes or molecules) as well as diverse states (such as post-translational modifications). A growing number of pathway databases and software tools provide BioPAX files as import or export formats (Shannon et al., 2003; Funahashi et al., 2008; Demir et al., 2010; Kelder et al., 2011) and BioPAX is useful to integrate information from heterogeneous sources, to support visualization, and analyses. The definition of BioPAX is the result of a continuous community effort. The BioPAX language is organized in levels that increasingly add features to the language definition. BioPAX is based on OWL and it is implemented as an ontology. An online validator can be used to check the correctness of BioPAX files. All elements within a BioPAX file can be annotated using controlled vocabularies and MIRIAM (Laible and Le Novère, 2007; Juty et al., 2012). For writing, reading, manipulating, and analyzing the API library Paxtools (Demir et al., 2013)^6^ has been created and is freely available. Quantitative relationships and temporal sequences of events do not belong to the objectives of BioPAX. However, since it is also possible to encode qualitative relationships in SBML (Chaouiya et al., 2013), BioPAX can be converted to SBML without loss of information (Büchel et al., 2012).

#### 2.2.5 NeuroML

The object-oriented mark-up language NeuroML (Gleeson et al., 2010)^7^ has been developed as a standard to specifically encode, share, and store computational models of information transfer in neurosciences (Goddard et al., 2001). The aim of the language is to cover diverse structural levels beginning at individual neuron cell membranes and ranging to entire neural networks. This XML-based language encodes biophysically detailed neuronal and network models including ion channels, synapses, and the anatomical connectivity of neurons and how these elements underlie the complex electrical behavior of the brain (Gleeson et al., 2010). Therefore, from the very beginning, modularity, portability, and clarity were the main language requirements (Goddard et al., 2001). Supporting high-performance simulations and creating software frameworks for neuroinformatics are the aims of the language (Beeman, 2013). To this end, NeuroML 2 has been built on the Low Entropy Model Specification (LEMS) language (Cannon et al., 2014), which hierarchically defines structure and dynamics of a large variety of biological models. For parsing, writing, and manipulating NeuroML and LEMS files, the Python APIs libNeuroML and PyLEMS as well as the Java™ APIs jNeuroML and jLEMS are available (Vella et al., 2014). The original idea to link sub-modules of processes in NeuroML to models encoded in SBML or CellML (Gleeson et al., 2010) has since been further elaborated. The LEMS libraries allow users to import SBML models and can also export SED-ML (Waltemath et al., 2011b) files for reproducible simulation experiments. The main repository for NeuroML is Open-Source Brain (Gleeson et al., 2013).

#### 2.2.6 ISML and PHML

The XML-based language ISML (*insilicoML*) allows users to describe biophysiological models that cross multiple scales and levels. This format is fully compatible to CellML 1.0, but incorporates a specific ontology of physiological functions (Asai et al., 2008). A large collection of models in ISML can be obtained from an online database at http://www.physiome.jp. The physiological hierarchy mark-up language (PHML) has been designed as the successor of ISML (Asai et al., 2013). PHML defines each biological or biophysical element as a module, which can be encapsulated and linked through ports. This concept hierarchically structures the language. Furthermore, PHML can integrate SBML models as sub-cellular phenomena (Asai et al., 2012).

#### 2.2.7 PharmML

The Pharmacometrics Mark-up Language PharmML (Moodie et al., 2013)^8^ belongs to the most recent languages in the family of XML-based standards for biomedical computation and is currently under development. The purpose of this new language is to exchange and store pharmacometric models, which includes studies, trials, simulations, estimation, and exploration. It will support metadata, non-linear mixed effects models, serve as an encoding platform for new approaches and elements, as well as support model-based analysis. The developers want to ensure backwards compatibility with existing relevant standards in order to use existing software tools. Use-case scenarios are, for instance, the kinetics of tumor growth, observation models, or trial design for treatment-dosing-related data.

#### 2.2.8 Synthetic biology open language

**Figure 2:**
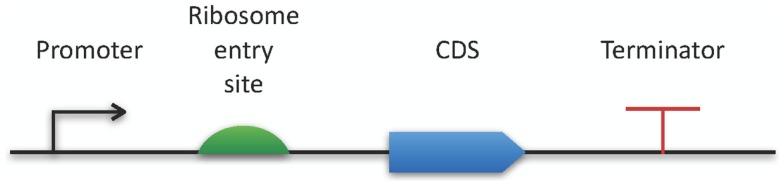
**SBOL visual**. The horizontal bar represents a DNA molecule to which various features can be visually attached. Here, a few examples are applied for demonstration purposes. A full specification and an exhaustive list of all available symbols can be found online at http://www.sbolstandard.org/visual.

The Synthetic Biology Open Language^9^ also belongs to the latest modeling standards (Galdzicki et al., 2014). This RDF-based format has been designed in a community process in order to facilitate the creation of synthetic biology components by providing an exchange format for software tools. As a specialty, SBOL comes with a specific graphical representation for promoters, their regulators, and many additional genetic structures (see Figure 2).

### 2.3 Standards for model simulation procedures

Defining the structure of a model does not give any information about reproducible simulation experiments. In order to perform the identical simulation of the model as described in a corresponding research article, the exact name of the numerical solving algorithm, step size, error tolerance, etc. must be precisely defined. The purpose of the Simulation Experiment Description Mark-up Language (SED-ML)^10^ is to provide a standardized, machine-readable, platform-independent data format for this purpose (Waltemath et al., 2011b). SED-ML follows the MIASE guidelines (Waltemath et al., 2011a) and hence enables users to attach both a model as well as the description of its intended use to a publication, which could also simplify review processes. It therefore contributes to the reproducibility aspect in science, where only stochastic approaches might diverge within a small range from published data. The XML-based language SED-ML is organized in levels and can describe multiple simulation experiments within the same file. Language components can be annotated using MIRIAM resources (Laible and Le Novère, 2007). A key idea of SED-ML is not to distribute concrete implementations of simulation procedures, but rather to use ontologies such as KiSAO (Courtot et al., 2011) to refer to the method and its settings. Since this ontology has a hierarchical structure, it is possible to apply related simulation algorithms in case a required method is not implemented in a certain software tool. Structural model changes prior to simulation and post-processing steps of the results (such as converting between amounts and concentration units) as well as the presentation of the output can also be defined (Waltemath et al., 2013). The model can, in principle, be encoded in an arbitrary standardized format and addressed through URI links. SED-ML does not provide an encoding of the simulation results itself, but can be used in combination with numerical mark-up language (NuML) or SBRGML (Dada et al., 2010). An extension to SED-ML has been proposed in order to also support sampling sensitivity analysis simulation experiments (Miller et al., 2012). Some simulation environments have already adopted this young format (Olivier et al., 2005; Myers et al., 2009; Kolpakov et al., 2011; Keller et al., 2013). A workflow editor (SED-ED), API libraries (libSedML, jlibSEDML), and a simplified scripting language (Antimony) are also available (Smith et al., 2009; Adams, 2012).

### 2.4 Graphical model representation formats

The visual representation of biochemical pathways has a long tradition. Displays of biological circuit diagrams and reaction pathways can be found in numerous textbooks and a plethora of publications. Databases such as KEGG (Kanehisa and Goto, 2000) or MetaCyc (Caspi et al., 2014) take this up and provide displays of biological networks in their specific layout and style, which follows many traditional aspects. In order to display and draw similar maps, several programs have been developed, for instance, CellDesigner (Funahashi et al., 2008), JDesigner (Sauro et al., 2003), TinkerCell (Chandran et al., 2009), VCell (Resasco et al., 2012), or Cytoscape (Shannon et al., 2003) with its diverse plug-ins (König et al., 2012; Gonçalves et al., 2013). We now discuss recommendations for the display of pathways and standardized data formats for exchanging these maps.

#### 2.4.1 SBGN and SBGN-ML

**Figure 3:**
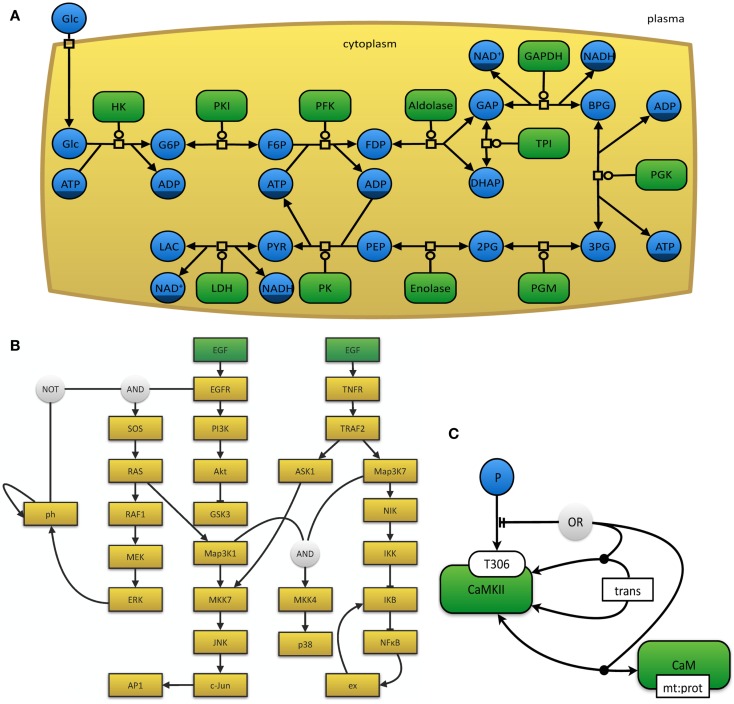
**(A)** The glycolysis in human erythrocytes, simplified from Dräger (2011). This example network depicts the reaction steps from extracellular glucose to intracellular lactose as a chain of enzyme-catalyzed reactions in SBGN PD notation. Metabolites that occur multiple times in the map, such as ATP or NAD**^+^**, have darker clone markers on the bottom. Simple molecules are displayed as circles, whereas, macromolecules appear as rounded rectangles. Reactions are indicated as square process nodes. **(B)** This activity flow diagram displays the interaction of two mammalian signaling pathways that are stimulated by epidermal growth factor (EGF) and tumor necrosis factor alpha (TNFα) and their influence on the nuclear factor κ-light-chain-enhancer of activated B cells (NFκB) and mitogen-activated protein kinases (MAPK) cascades. Adapted from Chaouiya et al. (2013) and generated with the program CellNOpt (Terfve et al., 2012). Here, external stimuli are colored in green. **(C)** This figure displays an example for an entity-relationship diagram, in which Ca^2+^/calmodulin-dependent protein kinase II (CaMKII) is precluded if it dimerizes or if it binds to the protein calmodulin. Adapted from Le Novere et al. (2011).

The myriad of graphical notations that are being used can lead to confusion or ambiguity. The development of a unified and standardized notation has thus become necessary (Le Novère et al., 2009). The Systems Biology Graphical Notation^11^ effort aims to make the display of biological networks exchangeable between software tools and at the same time to clearly define the meaning of specific nodes and arcs in such networks in order to ease their interpretation and automated processing. Therefore, the number of graphical symbols is intentionally limited in order to keep the learning curve flat and to create a visually, syntactically, and semantically consistent schema, which is modular in size and complexity (Le Novère et al., 2009). The SBGN neither defines layout (placement and adjustment) nor style (such as line thickness or color) of objects. In order to represent the current needs for such a display, it is organized in levels, so that in the future new versions can be proposed. The specifications of the SBGN are organized in three different languages, each of which has been designed for certain use-case scenarios and has inherent strengths and weaknesses. (i) In process-description diagrams (Kitano et al., 2005; Funahashi et al., 2008), the level of detail is very high and these maps show sequences of processes, which also involve temporal causality (see Figure 3A). These maps are well suited for metabolic pathways, but not for the consistent display of the combinatorial complexity of several proteins with many phosphorylation states (van Iersel et al., 2012). (ii) Activity flow charts (van Iersel et al., 2012) are much more abstract and neglect many molecular mechanisms. By design, these maps introduce a certain ambiguity and can hence be used to describe effects whose precise underlying mechanisms are either not know or not relevant (see Figure 3B). In this type of diagram, stimulation and inhibition, effects of perturbation, and the activity of components can be displayed. Activity flow charts are thus suitable for the display of causality chains (van Iersel et al., 2012). (iii) The entity-relationship diagrams (Kohn et al., 2006) are particularly useful when the temporal sequence of events does not play the main role, but precise molecular interactions are to be displayed (see Figure 3C). These maps are more concise than process-diagrams for protein modifications and interactions, but less capable of representing reactions (van Iersel et al., 2012).

In order to specifically store and exchange SBGN maps in XML files, the Mark-up Language SBGN-ML has been developed (van Iersel et al., 2012). The main requirement for this format is its simplicity, i.e., it should be easy to draw and to interpret. Most significantly, SBGN-ML is not tied to any of the network representation standards. While, this format does not include rendering information, it has been proposed to incorporate a rendering extension, similar as can be done with SBML files. In contrast to the SBML layout extension, this format is focused on the concepts of SBGN only and can be validated against the SBGN specifications. The API library libSBGN^12^ facilitates the import and export of SBGN-ML files. The code of libSBGN has been automatically created from an XML Schema Definition file (XSD), which significantly reduces the implementation effort, makes native language implementations in C++ and Java™ possible, and can be used for Schematron validation. A growing number of libSBGN-based software tools support the SBGN-ML format, such as the VANTED (Junker et al., 2006) plug-in SBGN-Ed (Czauderna et al., 2010), the Cytoscape (Shannon et al., 2003) plug-in CySBGN (Gonçalves et al., 2013), the online tool BioGrapher (Krause et al., 2013), the model generator KEGGtranslator (Wrzodek et al., 2013), or the visual editor CellDesigner (Funahashi et al., 2008).

#### 2.4.2 Visualization of CellML

For CellML, a specialized interactive framework has been developed for the display of models (Wimalaratne et al., 2009). This framework can either depict the physical model, i.e., the actual components of the CellML format, or the biological interpretation. CellML hence provides its own two-dimensional visual language for both concepts, which can be used in programs to link between the display and the underlying data structure, and also for dynamic image manipulation. For both kinds of displays, a small set of distinct glyphs are defined: entities, processes, and roles. While the physical display tends to be very complex, the biological view is much more straightforward. The developers of the CellML visualization scheme interact with the SBGN team (Wimalaratne et al., 2009). On the longer term, it is intended to combine ideas from SBGN (Le Novère et al., 2009) and the CellML display. Currently, not all concepts of the CellML display can be expressed in SBGN (Wimalaratne et al., 2009).

#### 2.4.3 Layouts in SBML

Layouts can directly be stored in SBML models with the help of the layout extension (Gauges et al., 2006). With this extension, it is possible to attach information about position and size of objects, such as reactive species, compartments, or reaction arcs. Text labels can also be placed. The SBML layout package is based on boundary boxes and defines neither shapes nor colors of objects, but it can be further extended with additional rendering information (Deckard et al., 2006; Shen et al., 2010). Tools such as SBML2LaTeX or SBML2Ti*k*Z (Dräger et al., 2009b; Shen et al., 2010) can interpret layouts stored in this extended SBML to be consistent with SBGN process-diagram maps. In general, these two SBML extensions allow users to store arbitrary forms of network representations. Programs such as KEGGtranslator (Wrzodek et al., 2011, 2013) use the layout extension to preserve initial layouts from the KEGG database in SBML files. In combination with the SBML extension for qualitative models (Chaouiya et al., 2013), it is also possible to create activity flow networks. In contrast to the SBML layout extension, no standardized way has been proposed to directly store SBGN-ML layouts inside of SBML files. However, the recent COMBINE format (Bergmann et al., 2014) allows users to store files of diverse forms all together within one archive file (see Section 2.6).

### 2.5 Representation of numerical results

In order to store the results of numerical simulation, specific file formats have been proposed. The Systems Biology Result Mark-up Language (SBRML, Dada et al., 2010) has been succeeded by the NuML^13^. This new format has been developed as a standardized exchange and archiving format for the results of numerical methods. This new language has been designed as a format that is usable in various disciplines besides systems biology. The C++ library libNUML can be used for parsing, manipulating, and writing the information of NuML data structures.

### 2.6 COMBINE format

The COMBINE format aims to distribute diverse modeling, documentation, and data files together within one single Open Modeling Exchange format (OMEX) file (Bergmann et al., 2014). The format is basically a ZIP archive, i.e., a compressed datatype, which contains an XML-based manifest file and an optional metadata file in RDF format. While the structure of the manifest file is well-defined, there are only recommendations for the metadata file. If present, metadata should at least include information about the author of the OMEX file in form of a vCard and follow the structure proposed by the Dublin Core Metadata Initiative. The manifest file contains structured links to all included files together with a definition URI that describes the filetype. Thus, diverse types of files can be included, even publications, plots, models, graph definitions, etc. Just for the sake of significant data compression, it is already recommended to store models inside of OMEX files (file extension *.omex). Even though the COMBINE archive format belongs to the most recent datatypes of the systems biology community, it is already supported by a number of tools and also the library libCombineArchive for dealing with it (Java™ and C#).

### 2.7 Online model repositories and databases

One important aspect of model exchange and reusability is the availability and distribution of models that have already been published or that are currently under review. Since a growing number of journals require the online availability of models along with a publication, it is important to be familiar with a number of online resources that are now available. In this section, we will discuss the different aims and features of selected online model repositories, which are summarized in Table 2.

**Table 2:** **Relevant online databases**.

Database	URL	Provides	Comments
BiGG	http://bigg.ucsd.edu	SBML	COBRA models
BioModels	http://www.ebi.ac.uk/biomodels-main/	CellML, SBML, PDF, VCML, and other formats	Main repository for SBML models
JWS	http://jjj.biochem.sun.ac.za/	JWS format, SBML	Online simulation facility
ModelDB	http://senselab.med.yale.edu/modeldb/	Various kinds of model data files	Focus on neuroscience
Open-source brain	http://www.opensourcebrain.org	NeuroML and PyNN	Interactive model development repository
PMR2	http://models.cellml.org	CellML	Project management platform with connection to JWS
SEEK	http://www.sysmo-db.org	Models in diverse formats, publications, and presentations	Focus on collaboration, connection to JWS
WikiPathways	http://www.wikipathways.org	BioPAX, PathVisio, and image formats	Interactive web 2.0 tool for biochemical pathways

#### 2.7.1 BiGG

An important resource for Biochemically, Genetically, and Genomically structured genome-scale metabolic network reconstruction is the BiGG database (Schellenberger et al., 2010). The main focus of this knowledge-base is to facilitate the bottom-up genome-scale reconstruction of metabolic networks. Inclusion of every known reaction of an entire organism constitutes the ultimate goal of BiGG. To this end, it integrates published genome-scale metabolic networks into one resource and applies a standard nomenclature for all of their components. Among these networks are several important model organisms, such as *E. coli* and *H. sapiens*, as well as further main branches of life (Duarte et al., 2007; Feist et al., 2007; Thiele et al., 2013). All models are manually curated and all reactions are atom-balanced. These networks also include gene–protein associations, which can be used to relate the activity of genes via Boolean logic to reactions and hence to perform knock-out or knock-down experiments *in silico*. BiGG offers various options to search, browse, and display networks. Manually curated maps can be downloaded in SVG format for a multitude of pathways. There are often several such maps available for one organism. Various build-in functions (such as decompartmentalization, orphan detection, gap filling, etc.) support the modeling process. With its SBML export function, it provides the basis for further steps in the modeling pipeline, particularly constraint-based analyses by the COBRA platform (Becker et al., 2007; Ebrahim et al., 2013). As the first database specific to constraint-based models, it precedes the SBML extension for fbc, but provides COBRA-specific model extensions that can be easily converted (Bornstein et al., 2008).

#### 2.7.2 BioModels database

BioModels database (Chelliah et al., 2013) is an open-source project, whose license model allows free commercial and academic use. Individual authors can submit their models to this database. A team of curators further improves the models, for instance by making the annotations in the model consistent with respect to MIRIAM guidelines (Juty et al., 2012). Large parts of the database content have been imported from collaborative repositories, such as the CellML model repository (Yu et al., 2011). The web interface of BioModels database provides a large variety of services based on embedded tools, e.g., for the simulation or graphical display of models. The main format of BioModels database is SBML, but models can be downloaded in a wide variety of formats, most of which have been automatically converted from the SBML files. It is also possible to obtain an exhaustive model report about each model (Dräger et al., 2009b) that describes the details of each model component in a human-readable way. Since the database was launched in 2005, it has been observed that not only are the number of models significantly increasing, but also their complexity. It now contains a large number of models, each describing the same biological process, but with higher levels of detail. With the growing size of the database the search for a model of interest has become a problem by itself (Schulz et al., 2011). With the help of metadata stored along with each model and the actual content of the models, sophisticated ranking procedures have been designed based on information theory aiming to retrieve models from the database for a given query (Henkel et al., 2010). The metadata include the submission and modification data, the authors of the model, and references. The user can browse through the models based on several characteristics, including the model name, publication identifier, or a GO-based (Ashburner et al., 2000) classification. Besides the curation of models, the main purpose of this repository includes the reproduction of model simulation results as given by the original publication (Waltemath et al., 2013).

#### 2.7.3 CellML physiome model repository 2

The CellML physiome model repository 2 (PMR2) is the most important resource for CellML models at different states of their curation (Yu et al., 2011). It uses a Plone-based model management system that is organized in workspaces. This allows its users to collaboratively develop models based on a version-control system and also facilitates the modular development of models. The models stored in this database cover a large variety of processes, including signal transduction and metabolic pathways, electro-physiological and cell cycle models, immunological models, and models describing muscle contraction or mechanical phenomena. The idea of collaborative model development brings with it one important feature: PMR2 keeps track of a detailed version history of all models. Plug-ins to the system facilitate the presentation of models in various ways and also enable the import and export of diverse modeling formats, including SBML or FieldML besides the native database format CellML. In addition, the plug-in technique makes the database extendable. A search function returns models of all curation states. The main focus of this database is to provide a version-controlled repository for the collaborative model development and presentation of model information, here called *exposures*.

#### 2.7.4 JWS online model repository

Another popular model resource is the JWS Online Model Repository (Snoep and Olivier, 2003). When JWS was launched as the first central model database in 2003 the standards SBML and CellML were still in their early development and not as well established. The repository itself is tightly related to the JWS online simulator (Olivier and Snoep, 2004), a particularly useful resource for educational purposes. Since then, the database has been continuously extended. Its native data format is SBML. Models can be queried based on a list of predefined characteristics (Waltemath et al., 2013), including metadata such as author, publication, organism, or model type as well as a list of categories (for instance, cell cycle or metabolism). The purpose of JWS is to provide a user-friendly online repository of kinetic models of biological systems in combination with an application that facilitates the simulation of these models. The aim of this infrastructure is to ease the review process of papers describing these kinds of models. As a result of its integration into the SEEK platform (Wolstencroft et al., 2011) a large number of collaborative projects use JWS as their default modeling platform.

#### 2.7.5 SEEK platform

The open-source SEEK platform benefits from the ability to offer JWS as its integrated simulation tool to its users. The SEEK platform goes beyond just being a model database. This web-based tool has been designed as a pragmatic data management solution for the exchange of very diverse kinds of data relevant for research in systems biology. Besides mathematical models, it also covers the exchange of experimental data, scientific protocols, and personal information about members of large research consortia (Wolstencroft et al., 2011). It allows its users to record the outcomes of experiments. One of its most important features is the ability to link between data, models, and publications, as well as to tag all uploaded items. This platform has originally been developed for the European SysMO consortium (Booth, 2007), and is also used in several other National and European projects, such as the German Virtual Liver Network (Holzhütter et al., 2012). The preferred modeling data format of SEEK is SBML with MIRIAM annotations.

#### 2.7.6 ModelDB

ModelDB (Migliore et al., 2003; Hines et al., 2004) belongs to the seven databases of SenseLab (NeuronDB, CellPropDB, ModelDB, Olfactory Receptor Database, OdorDB, OdorMapDB, and BrainPharm). SenseLab aims to provide a neural, genomics/genetics, proteomics, and imaging information resource for the neuroscience community and the interested public (Crasto et al., 2007). The database does not explicitly require a standard data format. Instead, authors are welcome to upload their models in arbitrary formats. As a result, the database is very flexible, but model reuse can take extra time to convert the desired model in a format for a particular execution environment (Waltemath et al., 2013).

#### 2.7.7 Open-source brain

Inspired by the open-source movement, the collaboration-oriented open-source brain (OSB) repository has been established (Gleeson et al., 2013). All models in this repository can be commented, debugged, and extended by registered users. This platform therefore complements repositories, such as ModelDB (Hines et al., 2004), which focus on distributing published models, with the aim to drive the advance of models at all stages of its development. An integrated WebGL-based 3D explorer allows users to view cells and networks in NeuroML 2 format within their browser. OSB is well integrated and links to ongoing research projects such as OpenWorm^14^.

#### 2.7.8 WikiPathways

The WikiPathways project (Kelder et al., 2011) provides a Web 2.0 wiki-based platform for the online curation of biological pathways. The idea for this platform is that manually curated pathways are of higher quality than automatically created ones. Motivating the scientific community to share knowledge would thus increase the quality of available pathway information. To this end, WikiPathways provides an interactive zoom-able pathway viewer that comes with a pathway diagram description, hyper-links, and detailed information as well as literature references. Users can also annotate the pathways with ontology terms. It is possible to submit private pathway information that is shared later with the public, for instance, as part of the review process, or if current knowledge about certain processes is limited. As a major feature, WikiPathways provides stable hyper-links to all pathways, which is useful in order to use the platform as a reference. Its content can be downloaded in many export formats under the terms of the Creative Commons license. The BioPAX standard (Demir et al., 2010) is thereby its most important format. Internally, it uses GPML, an XML standard that is compatible with many modeling tools, including Cytoscape (Shannon et al., 2003).

## 3 Results

### 3.1 Interoperability of standards

#### 3.1.1 Path2Models

An important driving force for improved interoperability and exchange of diverse data formats and standards was the community project path2models (Büchel et al., 2013a). The aim of this project was to automatically create draft models of biological processes based on the knowledge stored in the databases KEGG (Kanehisa and Goto, 2000), MetaCyc (Caspi et al., 2014), SABIO-RK (Wittig et al., 2014), and PID (Schaefer et al., 2009). The extraction of information from these databases required the development of new algorithms in order to capture a large variety of special cases (Wrzodek et al., 2011, 2013; Büchel et al., 2012) due to the different scope of the source databases. In order to also encode qualitative networks in SBML, the standard needed to be extended (Chaouiya et al., 2013). The draft SBML models had to be quality controlled and enriched with further kinetic information for reactions for which the SABIO-RK database did not yet provide experimentally determined rate laws (Dräger et al., 2008, 2010). Drafts of whole organism models were created by combining individual organism-specific pathway models (Swainston et al., 2011).

The main purposes of the KEGG databases are to provide a comprehensive, textbook-like educational view on the knowledge about a large variety of biological pathways. For modeling purposes, however, the information needs to be presented in a different way (Wrzodek et al., 2013). Reactions cannot be lumped together for the purpose of a better visual presentation, but have to be made explicit. The model must be as specific as possible, i.e., organism-specific variations must be reflected in pathways.

New algorithms also needed to be proposed in order to generate SBGN-ML files directly from KEGG (Czauderna et al., 2013). On the one hand, the manually created pathway maps in KEGG can be much better comprehended by human beholders than automatic layouts. However, in order to obtain an unambiguous representation of knowledge, the initial KEGG layout needs to be modified and subject to several constraints with respect to the esthetics of the result.

Such a large-scale endeavor, which resulted in more than 140,000 pathway maps that are all available from BioModels Database (Chelliah et al., 2013), was only feasible with the help of automatic procedures. Overall, this effort can be seen as a showcase application, which demonstrated the usefulness of data standardization, source code exchange, and software development in a large collaborative community project.

#### 3.1.2 Workbench and workflow approaches

**Figure 4:**
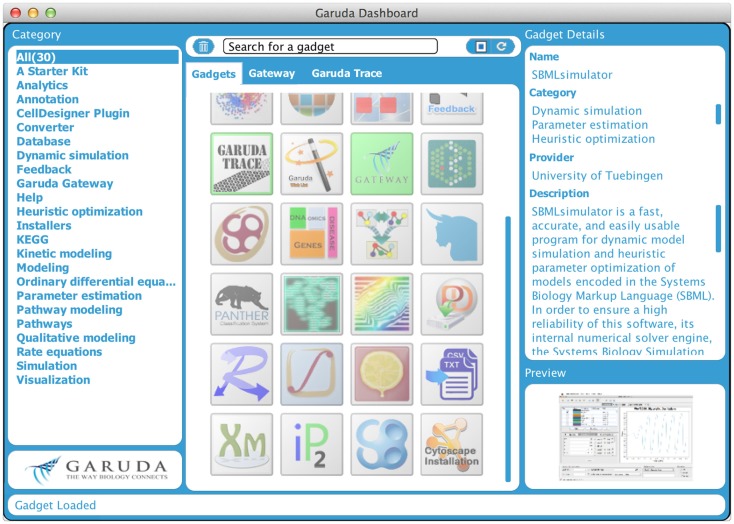
**Garuda dashboard**. This is the main screen of Garuda. The left column lists several categories that group individual gadgets. The icons in the center column allow users to launch applications with a double click. A detailed description of a gadget is displayed in the right column upon click on an icon.

Even though several data storage and exchange formats have been defined and software has been developed to import and export those formats, it is still difficult to work with a large number of different programs and in diverse environments. It can be of particular interest to process intermediate results from one program in another software package or to work with software on different computers with different operating systems. Furthermore, software is often written in diverse programing languages and compiled in diverse environments. Code reuse is still quite limited. All this can hamper building complex analysis pipelines. To address these problems, the systems biology workbench (SBW, Sauro et al., 2003) and the Garuda effort (Ghosh et al., 2011) have been launched. SBW is a software framework for communication between heterogeneous application components. It provides a broker to which each SBW-enabled software needs to register. This broker enables the software to be executed on different machines. Information can be sent from one program to the other through a specific protocol, which provides a fast binary encoded message system. SBW therefore allows programs to use each other’s capabilities. In contrast, Garuda is similar to an “App Store” for systems biology (see http://www.garuda-alliance.org/). It provides a common platform, from which diverse applications (gadgets) can be launched (see Figure 4). Garuda gadgets can call each other and send their output the next gadget or receive input from other gadgets. A powerful workflow would be to create a model with KEGGtranslator (Wrzodek et al., 2011, 2013), which can forward its result to the rate law generator SBMLsqueezer (Dräger et al., 2008, 2010), which in turn launches SBMLsimulator (Keller et al., 2013)in order to run a simulation and parameter calibration on the resulting model. Garuda provides a nice and easily understandable user interface, the dashboard, from which applications can be launched.

### 3.2 Software support

A large variety of software has been developed for many kinds of model building, analysis, drawing, simulation, and format interconversion. In this section, we will only discuss a small number of conceptual categories and particularly important tools. Several reviews specifically focus on available software (e.g., Dandekar et al., 2012; Hamilton and Reed, 2013; Fernández-Castané et al., 2014; Gostner et al., 2014; Koussa et al., 2014; Kramer et al., 2014). Table 3 gives an overview of selected software. For an up-to-date list and comprehensive information, see, for instance, the dynamic software matrix at http://sysbioapps.dyndns.org/pivot-software-matrix.html.

**Table 3:** **Selected relevant software for systems biology**.

Program	Main features	Citation
BiNA	Visualization of regulatory and metabolic network data with configurable styles and hierarchical graph concepts; analysis of omics data; data warehouse; plug-in system architecture	Gerasch et al. (2014)
BioGrapher	Web-based tool for creation and editing of SBGN maps with automatic layout algorithms	Krause et al. (2013)
BioUML	Platform for network building, simulation, analysis with full implementation of SBML	Kolpakov et al. (2011)
CellNOpt	Logic-based program for creating and simulating models of signal transduction	Terfve et al. (2012)
Cytoscape	Plug-in-based open-source software platform for visualizing complex networks and their attributes	Shannon et al. (2003)
CellDesigner	Process-diagram editor for gene-regulatory and biochemical networks with plug-in architecture and integrated solvers	Funahashi et al. (2008)
COBRA, COBRApy	Implementations of FBA, gene deletions, flux variability analysis, sampling, and batch simulations for constraint-based models	Schellenberger et al. (2011), Ebrahim et al. (2013)
COPASI	Simulation and analysis of biochemical networks and their dynamics in stochastic and ODE frameworks with support for SBW, parameter estimation, visualization, and several export formats	Hoops et al. (2006)
FASIMU	Command-line based collection of common FBA algorithms for SBML and several kinds of constraints. Its linear programing solvers can be exchanged and numerous constraints be defined	Hoppe et al. (2011)
Flint	An efficient stand-alone solver for PHML and SBML models, which also provides a cloud service	Asai et al. (2013)
GINsim	Simulator for qualitative gene interaction networks with graph-drawing capability, interactive user interface, and support for SBML qual	Gonzalez Gonzalez et al. (2006)
GRN2SBML	Converts the output of network inference procedures to SBML including MIRIAM annotation; access to BioMart central portal; R-package	Vlaic et al. (2013)
iBioSim	Modeling, analysis, design of genetic circuits for systems, and synthetic biology; user-friendly editors for diverse formats; variety of ODE and stochastic simulators; and plotting functions	Myers et al. (2009)
JSim	Building and analysis of quantitative numeric models with focus on physiology and biomedicine; support for ODEs, PDEs, implicit equations, etc.	Butterworth et al. (2014)
libRoad-Runner	C++ library for efficient numerical simulation and analysis of SBML models that provides Python language-bindings, which are integrated into the tellurium environment	Sauro et al. (2013)
libSBMLSim	C-based ODE simulation library for SBML models with explicit and implicit methods, language-bindings, and command-line tool	Takizawa et al. (2013)
Mass-Toolbox	Mathematica framework for kinetic and constraint-based model building and simulation; focus on mass-action kinetics and elementary reaction systems; support for ODE/DAE (incl. delays and events)	Sonnenschein and Palsson (2013)
Module-Master	Identification of *cis*-regulatory modules (CRMs) in sets of co-expressed genes based on transcription factor binding information and multivariate functional relationships between regulators and target genes	Wrzodek et al. (2010)
MOOSE	Multi-scale object-oriented simulation environment for diverse biological systems with a Python scripting interface and support for SBML, NeuroML, GENESIS kkit, and cell.p formats	Dudani et al. (2013)
OpenCOR	Plug-in based cross-platform modeling environment for working with CellML files	Nickerson et al. (2013)
Physio-Designer	Platform for the creation and analysis of PHML models that also allows users to integrate SBML models. It uses Flint as its solver back-end through a cloud service	Asai et al. (2013)
PySCeS	Extendable Python toolbox for time-course simulation, steady-state and stability analysis, metabolic control analysis and many more, support for SBML fbc and SED-ML	Olivier et al. (2005)
SOSlib	C programing library for symbolic and numerical analysis of chemical reaction network models encoded in SBML format	Hindmarsh et al. (2005), Machné et al. (2006)
SBML-simulator	Dynamic model simulation and heuristic parameter optimization of SBML models based on the systems biology simulation core library and EvA2	Kronfeld (2008), Keller et al. (2013)
SBML-squeezer	Context-sensitive generator for kinetic equations of biochemical and gene-regulatory networks with access to SABIO-RK	Dräger et al. (2008, 2010), Dräger (2011)
SBToolbox2	MATLAB™ toolbox with support for SBML, and a large variety of analysis and high-performance simulation functions as well as parameter estimation, sensitivity analyses	Schmidt and Jirstrand (2006), Schmidt (2007)
TinkerCell	Computer-aided design platform for synthetic biology with C and Python API	Chandran et al. (2009)
VANTED	Versatile plug-in based visualization and analysis platform for networks with support for SBGN-ML, sophisticated layout algorithms, and FBA	Junker et al. (2006)
VCell	Modeling and simulation (deterministic and stochastic) of physicochemical and electro-physiological processes with support for irregular spatial distribution of substances in arbitrary geometries	Moraru et al. (2008), Resasco et al. (2012)

#### 3.2.1 Visualization and model building

Several tools provide interactive graph-based user interfaces and facilitate import or creation, manipulation, or export of complex pathway structures. Some programs can be extended via plug-ins, e.g., the Biological Network Analyzer BiNA (Gerasch et al., 2014), CellDesigner (Funahashi et al., 2008), or Cytoscape (Shannon et al., 2003). The flexible stand-alone application BiNA (Gerasch et al., 2014) is based on a hierarchical graph concept and provides highly configurable styles for the visualization of regulatory and metabolic network data as well as access to the BN++ pathway data warehouse (Küntzer et al., 2007). The web-modeling tool BioGrapher (Krause et al., 2013) is implemented with HTML5, CSS, and JavaScript and can be used to create SBGN maps. BioGrapher can import several standard formats, including SBML and SBGN-ML, and export SBGN maps in a JSON file format or as images. The VANTED plug-in SBGN-ED supports all three kinds of SBGN maps and is therefore useful for designing and modifying SBGN-ML files (Czauderna et al., 2010). The framework program Cytoscape supports creation, import, and export of SBML and SBGN through plug-ins (König et al., 2012; Gonçalves et al., 2013). The main purpose of the straightforward and user-friendly process-diagram editor CellDesigner is the creation, manipulation, and simulation of SBML models (Matsuoka et al., 2014) with export functions to BioPAX (Mi et al., 2011) and SBGN-ML (van Iersel et al., 2012). CellDesigner can be extended through plug-ins, such as the kinetic law generator SBMLsqueezer (Dräger et al., 2008, 2010). The draft model generator KEGGtranslator (Wrzodek et al., 2011, 2013) automatically downloads contents of the pathway database KEGG (Kanehisa and Goto, 2000) and converts the content to diverse output formats, including SBML with extensions for layout (Gauges et al., 2006) and qual (Chaouiya et al., 2013), SBGN-ML (van Iersel et al., 2012), BioPAX (Demir et al., 2010), and many more. TinkerCell (Chandran et al., 2009) has been developed as a computer-aided design (CAD) tool and provides visual representations for systems biology and synthetic biology. OpenCOR (open-source cross-platform) for working with CellML files can be used through command-line or graphical user interface (Nickerson et al., 2013). It supports various aspects of modeling, including editing, simulation, and analysis. As a plug-in based program, OpenCOR can be easily extended. One of its most recent plug-ins facilitates the annotation of CellML.

#### 3.2.2 Constraint-based modeling

The most important toolboxes for Constraint-Based Reconstruction and Analysis (Bordbar et al., 2014) are the COBRA Toolbox for MATLAB (Schellenberger et al., 2011) and its Python implementation COBRApy (Ebrahim et al., 2013). These toolboxes provide state-of-the-art implementations of flux balance analysis methods, including gene deletions, flux variability analysis, sampling, and batch simulations. Both versions of COBRA incorporate tools to read-in and manipulate constraint-based models, which requires a specific extension of the SBML standard. The Mathematica-based Mass-Toolbox (Sonnenschein and Palsson, 2013)^15^ is a complex framework for constraint-based model building and simulation, which can calculate steady-state solutions for complex enzyme reactions and even solve ODE and DAE systems with delays and events. Further important tools for FBA are FASIMU (Hoppe et al., 2011), the VANTED (Junker et al., 2006) plug-in FBA-SimVis (Grafahrend-Belau et al., 2009), and PySCeS (Olivier et al., 2005).

#### 3.2.3 Dynamic simulation

The main focus of the Mass-Toolbox (Palsson, 2011; Sonnenschein and Palsson, 2013) is kinetic modeling with a focus on mass-action rate laws and elementary reaction systems. It supports a large variety of analysis methods and high-level plotting commands for phaseportraits, and many more.

The SBToolbox2 (see http://sbtoolbox2.org, Schmidt and Jirstrand, 2006; Schmidt, 2007) provides a powerful and extensible variety of simulation and analysis functions, which smoothly integrate into the MATLAB environment. SBToolbox2 supports SBML and parameter estimation with EvA2 (Kronfeld, 2008).

CellDesigner delivers several third-party tools for interactive model simulation SOSlib (Machné et al., 2006), the Simulation Core Library (Keller et al., 2013), or COPASI (Hoops et al., 2006).

The SBW-enabled complex pathway simulation program COPASI is primarily a stand-alone program, but provides API language-bindings for several programing languages. COPASI can read, write, and understand SBML, but has its own specific modeling language and supports several other export formats. It comprises methods for simulation and analysis of biochemical networks and their dynamics based on ODEs and stochastic systems. Parameter estimation and the visualization of data as well as animated pathways are among its strengths.

The tool SBMLsimulator combines the Simulation Core Library, a comprehensive Java™ API for solving SBML models (Keller et al., 2013) with the optimization framework EvA2 (Kronfeld, 2008) in a self-explanatory user interface and provides a complete implementation of the SBML standard in terms of an ODE framework.

The stand-alone desktop tool BioUML (Kolpakov et al., 2011) is among the few tools that provide a full implementation of the SBML standard in terms of ODE systems and also provides its functions as JavaScript API.

The stand-alone tool iBioSim (Myers et al., 2009) for modeling, analysis, and design of genetic circuits has been developed as an editor and simulator (ODE and stochastic) with applications in systems biology as well as synthetic biology. Besides SBML, it also understands Petri net (LPN) models and has import access to model databases. Experimental data can also be used to infer models in iBioSim.

SOSlib (Machné et al., 2006) is an ODE-based C-API library implementation of SBML that internally uses CVODE (Hindmarsh et al., 2005). The newer C-implementation libSBMLSim (Takizawa et al., 2013) supports even more recent versions of SBML, explicit and implicit integration methods, and bindings to several programing languages. Another alternative is libRoadRunner, a highly performant C++ library for the simulation of SBML models, which provides automatically generated language-bindings to Python (Sauro et al., 2013).

The Java-based tool JSim has been designed for building quantitative numeric models as well as the analysis of these models based on given experimental data (Butterworth et al., 2014). It supports ODEs and PDEs, discrete events, and implicit methods. JSim can import and export SBML and import CellML (Smith et al., 2013a).

The Virtual Cell suite VCell is a powerful simulation toolbox for complex biological phenomena (Moraru et al., 2008; Resasco et al., 2012). It includes sophisticated methods for: (i) molecular interactions and transport, (ii) various sub-cellular compartments, (iii) dynamics of membrane potentials, and (iv) arbitrary fluxes and passive cross-membrane transport mechanisms, and supports PDEs in addition to ODEs. It is one of very few tools to incorporate physicochemical and electro-physiological processes and can apply quasi-steady-state approximations to fast reactions. It is also an image processing tool for experimental images.

The simulation environment MOOSE was developed as a reimplementation of the GENESIS neural simulator, and initially used that simulator’s model description format. Recently though it has developed support for NeuroML models, and is also capable of dealing with systems biological models (Gleeson et al., 2010; Dudani et al., 2013). New simulation algorithms can be added to MOOSE through a generic framework. It has also been developed with a focus on multi-scale models and simulation in diverse levels of detail (Dudani et al., 2013). For a more comprehensive overview about recent simulation tools with a focus on neuroscience we refer the interested reader to the review by Gleeson (2013).

The stand-alone modeling framework PhysioDesigner (Asai et al., 2013) provides several functions for the creation and analysis of PHML models. SBML models can be incorporated as submodels through PhysioDesigner (Asai et al., 2014), aiming at integrating dynamics at sub-cellular and cellular levels. The simulator Flint can efficiently solve PHML models and provides a cloud service, which allows users to remotely solve their models (Asai et al., 2012). PhysioDesigner uses Flint and submits jobs to this cloud service.

#### 3.2.4 Regulatory networks

The inference of regulatory networks is a challenge for many areas of research. The program ModuleMaster (Wrzodek et al., 2010) identifies *cis*-regulatory modules (CRMs) in sets of co-expressed genes based on transcription factor binding information and multivariate functional relationships between regulators and target genes. As an input it uses microarray and clustering experiments and SBML models as output. In order to make the results of network inference procedures such as Net*Gene*rator (Töpfer et al., 2007) reusable in further analysis tools, the program GRN2SBML (Vlaic et al., 2013) has been developed as a converter to SBML. It provides a graphical user interface, access to BioMart Central, and can also be used as an R-package. The program GINsim has been developed for the analysis and simulation of logical models of gene interaction networks (Gonzalez Gonzalez et al., 2006) and has been recently adapted to the SBML qual extension (Chaouiya et al., 2013). The program CellNOpt can be useful for the creation of signal transduction networks based on a logical approach (Terfve et al., 2012), and it also supports SBML qual.

### 3.3 Regular community meetings

Many standards described in this paper are based on community efforts. For this reason, community meetings have been required from their inception. In October 2010, separate workshops were combined in order to better coordinate individual developments and to reduce the necessary amount of traveling for individual researchers. This resulted in two regular annual meetings that brought together the community. The COMBINE (Computational Modeling in Biology Network) is a workshop with scientific presentations, poster sessions, and several break-out sessions, which are used to discuss and coordinate the further development of the “COMBINE Standards” BioPAX, CellML, SBGN, SBML, SBOL, and SED-ML, as well as associated and related standards. The idea of the spring Hackathon on resources for modeling in biology (HARMONY) is to provide room and time for community members to sit down, share code and ideas, program, and discuss. In contrast to the fall event, HARMONY usually has only very few talks and is much more a hands-on practical event, where participants develop new approaches and ideas. For more information about previous meetings see the meeting reports by Le Novère et al. (2011), Waltemath et al. (2014) and the COMBINE homepage^16^. This alternating sequence of complementary meetings leads to a very efficient and progressive development of software and standards.

## 4 Discussion

In this review article, we have examined diverse modeling standards and data formats that are currently in use within the scientific community together, with databases from where these formats can be obtained. We discussed a selection of useful software packages and modeling approaches for systems biology and related fields. The structuring of individual standards is at present very elaborate: there is usually a modeling, annotation, or documentation recommendation that forms the theoretical basis for a corresponding machine-readable data format and involves specific controlled vocabulary terms for unambiguous specification of individual model components.

Aiming to keep even highly elaborate standards flexible and able to incorporate new findings, the specifications are becoming more and more abstract and modularized. For example, the original *reaction* element in SBML is now seen as a generic *process* whose inputs and outputs no longer strictly have to *represent* substrates and products of biochemical reactions. The idea to develop specific packages for certain needs rather than one monolithic modeling language also follows this trend. The development of all standards involves numerous people, detailed discussions, and careful consideration. This overall procedure ensures that standards mature in an open fashion and allows interested researchers to participate and to contribute to this development. At the same time, it also increases the chance that potential conflicts or inaccuracies can be discovered in early stages of development. With increased use of standards the requirements of the individual format are steadily improved and current limitations are detected and solved. Thanks to the regular meetings and ongoing exchange between the developers of the diverse standards, the individual formats are mutually adopting more and more of each other’s features. It can therefore be expected that the exchange between different model and pathway representation standards will further increase.

For end-user applications, the goal is that users would no longer have to care about the underlying data format used by a specific software tool. More and more details of the internal structure and organization of underlying formats could be hidden and no detailed knowledge about these formats would be required. Plug-ins for platforms such as Cytoscape (Shannon et al., 2003) or CellDesigner (Funahashi et al., 2008) can provide complementary functionality for export or import of certain data formats based on a common underlying data structure (König et al., 2012; Gonçalves et al., 2013). The SBW or the Garuda framework provides further ways to increase the interoperability of tools with little effort (Sauro et al., 2003; Ghosh et al., 2011). Many tools could also benefit from the ability of the new COMBINE archive format to bridge separately stored representations or applications of the same model (Bergmann et al., 2014).

The distribution and curation of standardized models, their simulation description, and expected results by centralized databases plays a prominent role. These knowledge bases constitute valuable resources of available information about biological processes and reproducible experiments. They can therefore significantly reduce time and effort needed for the assembly of extended models and create the basis for further research. The ability to easily reproduce new scientific findings with existing simulation workflows facilitates the fast adoption and integration of these findings into new and even further elaborated works. If other researchers are able to run simulations and to comprehend models with minimal effort, it can be expected that these studies will receive higher recognition and lead to more citations compared to distributing models whose outcomes are difficult to reproduce. The distribution of models and data in standard formats amongst their project working groups will not only benefit collaboration partners, but the fine-grained structure of standards for diverse aspects of modeling workflows that is now available can even simplify the review process of scientific papers. If a model is uploaded along with a publication in a standard format, accompanied with a simulation experiment description file and a graphical representation, reviewers can quickly obtain an overview about structure and organization of a model, and even easily check if the findings described in the paper can be reproduced. Thereby, the reviewer can select any numerical tool that supports these data formats and is not restricted to any particular environment.

The development of a standard can be seen as a long-term investment. Unlike in other fields, the community-based bottom-up development of exchange formats is very common in systems biology. Depending on the structure of the field, it can therefore take a long time before the overhead of developing a new standard pays off; on the other hand, standards exist as long as the community has a requirement for them (Brazma et al., 2006). It also seems that the development of standards has become a field of research by itself and is sometimes even seen as the central aspect in modeling (Waltemath et al., 2013). Models and their evaluation are certainly valuable tools for progress in research, but permanently keeping track of all emerging standards can become difficult. The proposed concepts and approaches can only be successful if these are well-known. If standard data formats are developed that are not adopted by the community, the standard will disappear and a simpler solution will gain acceptance. As we go along, new modeling techniques and new finding are established and adopted by the research community (Lerman et al., 2012; O’Brien et al., 2013). Approaches for model encoding and standardization therefore need to continuously evolve with the domain of research that they represent. It is therefore important for the standardization community to continue to closely interact with the modeling community in order to catch up with novel approaches, needs, and requirements. The solutions given to the modeling community must be simple enough in order to be easily adopted, implemented, and applied, but they must also be sophisticated enough in order to capture the complexity of the described systems. Participation of the community in proposing encoding schemes and guideline checklists is essential for the success of the respective standard. Large-scale reconstructions and community projects require data standards and at the same time push their development (Büchel et al., 2013a; Thiele et al., 2013).

While in the past even quick computation in active research required the implementation of some data structures from scratch in customized scripts, the rich variety of software libraries and modeling-specific scripting languages now available drastically simplify these tasks. If an existing software solution cannot be directly applied to solve a specific task, it is at least possible to use standards compliant data structures from the very beginning of a project. Also the quality of available software solutions is progressively increasing. For the distribution of final results, standard formats should be used as the preferred exchange and storage medium in order to ensure reusability and reproducibility of results and findings.

## Author Contributions

Andreas Dräger developed the conceptual idea for this review and drafted the manuscript. Bernhard Ø. Palsson supervised this work and critically revised the manuscript. Both authors approve the final manuscript.

## Conflict of Interest Statement

The authors declare that the research was conducted in the absence of any commercial or financial relationships that could be construed as a potential conflict of interest.

## Footnotes

^1^http://obofoundry.org

^2^http://sbml.org

^3^http://www.cellml.org

^4^http://physiomeproject.org/software/fieldml

^5^http://www.biopax.org

^6^http://www.biopax.org/paxtools/

^7^http://www.neuroml.org

^8^http://www.ddmore.eu

^9^http://www.sbolstandard.org

^10^http://sed-ml.org

^11^http://sbgn.org

^12^http://libsbgn.sourceforge.net

^13^http://code.google.com/p/numl/

^14^http://openworm.org

^15^http://opencobra.github.io/MASS-Toolbox/

^16^http://co.mbine.org

## Abbreviations

ANSI, American National Standards Institute; API, application programing interface; BRAIN, brain research through advancing innovative neurotechnologies; CAD, computer-aided design; COPASI, complex pathway simulator; CSS, cascading style sheets; DAE, differential-algebraic equation; DIN, Deutsches Institut für Normung; FBA, flux balance analysis; fbc, flux balance constraints; GO, gene ontology; HTML, hyper text mark-up language; IEEE, Institute of Electrical and Electronics Engineers; IETF, internet engineering task force; ISML, *in silico* mark-up language; JSON, JavaScript object notation; KiSAO, kinetic simulation algorithm ontology; LEMS, low entropy model specification; MAMO, mathematical modeling ontology; MIASE, minimum information about a simulation experiment; MIBBI, minimal information for biological and biomedical research; MIRIAM, minimal information required in the annotation of models; NCBI, National Center for Biotechnology Information; NuML, numerical mark-up language; OBO, open biomedical ontologies; ODE, ordinary differential equation; OMEX, open modeling exchange format; OMG, object management group; OSB, open-source brain; OWL, web ontology language; PDE, partial differential equation; PharmML, pharmacometrics mark-up language; PHML, physiological hierarchy mark-up language; RDF, resource description framework; SBGN, systems biology graphical notation; SBGN-ML, systems biology graphical notation mark-up language; SBML, systems biology mark-up language; SBOL, synthetic biology open language; SBRML, systems biology result mark-up language; SBW, systems biology workbench; SED-ML, simulation experiment description mark-up language; SVG, scalable vector graphics; SWIG, simplified wrapper and interface generator; TEDDY, terminology for the description of dynamics; URI, uniform resource identifier; W3C, world wide web consortium; XML, eXtended mark-up language.
